# Revisiting the hypothesis of syndromic frailty: a cross-sectional study of the structural validity of the frailty phenotype

**DOI:** 10.1186/s12877-020-01839-7

**Published:** 2020-10-27

**Authors:** François Béland, Dominic Julien, Christina Wolfson, Howard Bergman, Pierrette Gaudreau, Claude Galand, John Fletcher, Maria-Victoria Zunzunegui, Bryna Shatenstein, Marie-Jeanne Kergoat, José A. Morais, Tamàs Fülöp

**Affiliations:** 1 Groupe de recherche Solidage, Lady Davis Institute, Jewish General Hospital, 3755 Chemin-de-la-Côte-Ste-Catherine, Montréal, Québec, H3T 1E2 Canada; 2 Département de gestion, d’évaluation et de politique de santé, École de santé publique, Université de Montréal, C.P. 6128, Succursale Centre-Ville, Montréal, Québec, H3C 3J7 Canada; 3 Centre de recherche en santé publique, Université de Montréal, C.P. 6128, Succursale Centre-Ville, Montréal, Québec, H3C 3J7 Canada; 4 Department of Epidemiology, Biostatistics and Occupational Health, Faculty of Medicine, McGill University, 1020 Avenue des-Pins-Ouest, Montréal, Québec, H3A 1A2 Canada; 5 Department of Family Medicine, McGill University, 5858, chemin de la Côte-des-Neiges, Suite 300, Montreal, Québec, H3S 1Z1 Canada; 6 Départment de médecine, Faculté de Médecine, Université de Montréal, C.P. 6128, Pavillon R, Salle R05-436, Montréal, Québec, H2X 0A9 Canada; 7 Centre de recherche du Centre hospitalier de l’Université de Montréal, 900 rue St-Denis, R Pavillon, Room R05-436, Montréal, Québec, H2X 0A9 Canada; 8 Département de Médecine sociale et préventive, École de santé publique, Université de Montréal, C.P. 6128, Succ. Centre-Ville, Montréal, Québec, H3C 3J7 Canada; 9 Département de nutrition, Faculté de médecine, Université de Montréal, C.P. 6128, succ. Centre-Ville, Montréal, Québec, H3C 3J7 Canada; 10 Centre de recherche, Institut Universitaire de Gériatrie de Montréal, CIUSSS du Centre-sud-de-l’Île-de-Montréal, Montréal, QC H3W 1W5, 4565, Chemin-de-la Reine-Marie, Montréal, Québec, H3W 1W5 Canada; 11 Division of Geriatric Medicine, MUHC-Montreal General Hospital, Room E-16.124.1, 1650 Avenue Cedar, Montréal, Québec, H3G 1A4 Canada; 12 CSSS-IUGS, Pavillon Argyll, Université de Sherbrooke, 375, rue Argyll, Sherbrooke, Québec, J1H 3H5 Canada

**Keywords:** Frailty, Syndrome, Phenotype, Latent class analysis, Factor mixture models

## Abstract

**Background:**

Fried’s Phenotype Model of Frailty (PMF) postulates that frailty is a syndrome. Features of a syndrome are a heterogeneous population that can be split into at least two classes, those presenting and those not presenting the syndrome. Syndromes are characterized by a specific mixture of signs and symptoms which increase in prevalence, from less to more severe classes. So far, the null hypothesis of homogeneity – signs and symptoms of frailty cannot identify at least two classes – has been tested using Latent Class Analysis (LCA) on the five dichotomized components of PMF (unintentional weight loss, exhaustion, weakness, slowness, and low physical activity). The aim of this study is to investigate further the construct validity of frailty as a syndrome using the extension offered by Factor Mixture Models (FMM).

**Methods:**

LCA on dichotomized scores and FMM on continuous scores were conducted to test homogeneity on the five PMF components in a sample of 1643 community-dwelling older adults living in Québec, Canada (FRéLE).

**Results:**

With dichotomized LCA, three frailty classes were found: robust, prefrail and frail, and the hypothesis of homogeneity was rejected. However, in FMM, frailty was better represented as a continuous variable than as latent heterogeneous classes. Thus, the PMF measurement model of frailty did not meet the features of a syndrome in this study.

**Conclusion:**

Using the FRéLE cohort, the PMF measurement model validity is questioned. Valid measurement of a syndrome depends on an understanding of its etiological factors and pathophysiological processes, and on a modelling of how the measured components are linked to these processes. Without these features, assessing frailty in a clinical setting may not improve patient health. Research on frailty should address these issues before promoting its use in clinical settings.

**Supplementary Information:**

The online version contains supplementary material available at 10.1186/s12877-020-01839-7.

## Background

The Fried Phenotype Model of Frailty (PMF) [1, 2] is characterized by cumulative decline across multiple physiologic systems resulting in decreased reserve and resistance to stressors, leading to increased risk for adverse outcome [2]. Five components are used as an index in the 5c-PMF measurement model (5c-PMF refers herein to the measurement model of the PMF): unintentional weight loss, exhaustion, weakness, slowness, and low physical activity. Based on the number of components, individuals are categorized as robust (no component), prefrail (1–2 components), or frail (≥ 3 components) [2]. The PMF 3-class model hypothesizes [2, 3] that individuals are homogeneous within each frailty class, but heterogeneous between classes.

The 5c-PMF measurement model, based on a model of the cycle of frailty from which syndrome components were identified, is one of the most widely used instruments in clinical practice [4] and can be considered foundational in the biological approach to frailty. Structural validity of the 5c-PMF was studied, and hypotheses and statistical procedures suggested. Compared to many frailty instruments, the PMF has been extensively validated. Most instruments measuring frailty were validated only as risk assessment tools [4–6].

In the PMF, frailty is conceptualized as a medical syndrome [2, 3, 7] which is a set of interwoven components [8] presenting two defining features. First, a syndrome is a manifestation of a phenotype. A population is hypothesized to be heterogeneous with at least one class of individuals presenting a significant number or all of its components, and at least one class that does not. Second, if frailty is a syndrome, frailty components are expected to aggregate within classes of a Latent Class Analysis (LCA) according to a similar gradient [3]. That is, the prevalence of frailty components are expected to increase progressively from robust to frail classes. If they co-occur, that is, if subsets of criteria occur preferentially in some of the classes only, then frailty is the result of distinct biological processes, rather than the expression of a single phenotype. Different syndromes may share subsets of components, but are differentiated from each other by a specific grouping of components.

These two features have been tested using LCA in several studies [3, 9–11]; all rejected the null hypothesis of K = 1-class model (homogeneity) and concluded that the 3-class heterogeneity model was the best-fitting model. However, in some of these studies, the 3-class and the 2-class models did not differ statistically using the Bayesian Information Criterion (BIC) and the chi-square goodness of fit test. Lohman et al. [11] rejected the 2-class model based on the Lo-Mendell-Rubin test. In addition, Liu et al. [10] could not reject the 4-class model.

Previous studies investigated frailty classes based on a K = 1 null hypothesis. However, rejecting K = 1-class models is different than accepting K > 1-class models. The hypothesis of similar manifestations of components among frailty classes can be tested by comparing a K > 1-categorical approximation of a continuous process for frailty (homogeneity) against the hypothesis of a “true K > 1-class” model (heterogeneity). In the former case, the classes are no more than recoding of a continuous variable over K homogeneous categories – classification parameters are equal across classes [12]. None of the previous studies tested this null hypothesis.

Most studies investigating frailty with a priori dichotomized continuous component scores are based on adaptations of the Bandeen-Roche et al. [3] procedures. Prior dichotomization of component scores is justified on the basis that the frailty syndrome classifies individuals in at least two classes: those that present and those that do not present the syndrome [3]. However, dichotomization is a characteristic of the syndrome, not of the components of the syndrome. A priori dichotomization of scores assumes that frailty is a syndrome, before testing the hypothesis of heterogeneity of a population on the components of frailty.

The aim of this study was to investigate the construct validity of frailty as a syndrome, using Factor Mixture Models (FMM) [13] on continuous frailty component measures. Three null hypotheses stemming from the Bandeen-Roche et al. [3] theoretical framework are tested: 1. K > 1-categorical representation of a continuous process; 2. K = 1-class; and 3. frailty components are ordered differently among classes, implying multiple etiological and pathophysiological processes.

## Methods

### Sampling frame

Participants were from the FRéLE study (*Fragilité: une étude longitudinale de ses expressions*). Three databases [14–16] were used to estimate the distribution of frailty as in the PMF for sample size calculation. Results showed that six equal size strata (men and women in three age groups: 65–74, 75–84, and ≥ 85 years old), each with 270 respondents, were appropriate to identify frailty classes [17].

The *Régie de l’assurance maladie du Québec* (RAMQ - Québec public and universal health insurance program agency) database was used to select randomly the FRéLE sample (*n* = 1643). Community-dwelling adults, aged 65 or older, were recruited in 2010 from three areas: metropolitan (Montréal), urban (Sherbrooke), and urban-rural (Victoriaville). Some of the FRéLE baseline questions were selected from the Canadian Community Health Survey (CCHS) [18]. Comparison of the Québec CCHS and FRéLE respondents showed that FRéLE reflects basic socio-economic and health status characteristics of the elderly population across Québec [19].

The sampling frame has been described previously [17, 19]. Three panels were collected over two years. Of the 1643 respondents at baseline (T0), 84.4% participated at T1 and 88.4% at T2. Losses were due to mortality (13% over the data collection period), or to voluntary dropout and inability to locate (13%). Construct validity of the 5c-PMF measurement model was examined with T0 data only. Predictive validity of the frailty classes was estimated with T2 data and mortality over a three-year period after T0.

### Measures

The five components of the PMF were assessed (Table 1) with a mix of performance tests and self-reported questions. Each measure is extensively described in Provencher et al. [19]. Self-reported unintentional weight loss of 10% of normal weight or lost of 4.5 kg or more during the previous year were used to assess the 5c_PMF *weight loss* component. The “vitality” section of the SF-36 [20] was used to assess *exhaustion*. The PASE [21] (Physical Activity Scale for the Elderly), a brief and valid instrument for measuring physical activity in elderly population, was used to measure *low physical activities*. Time to walk, from a stationary position, a distance of 2.44, 3 or 4 m, according to the space available at the participant’s home [22], was used to assess *slowness*. Finally, weakness was obtained from grip strength measured on a Martin Vigorimeter, using the American Society of Hand Therapists protocol [22].

**Table 1:** Frailty Components: Cut-points for the CHS and the FRéLE data sets

CHS Definition*	CHS Sample* (%)	WHAS Sample* (%)	FRéLE Definition	FRéLE Sample (%)
**Weight loss**	7,3%	12,7%	**Weight loss**	13.3%
Lost > 10 ponds in last year			Self-reported unintentional weight loss of 10% of normal weight or 4.5 kg or more.	
**Exhaustion**	21.3%	14,1%	**Exhaustion**	20.1%
Self-Report of either of:			The “vitality” subscale of the SF-36:	
i) Felt that everything I did was an effort in the last week, or			First quintile at 46.88	
ii) could not get going in the last week				
**Low energy**	24,1%	19.8%	**Low energy**	20.4%
270 on activity scale (18 items)			Physical Activity Scale for the Elderly	
			First quintile at 32.33 for women	
			First quintile at 39.35 for men	
**Slowness**	38,0%	31.3%	**Slowness**	20.9%
Walking 15 ft (4.57 m):			Guralnik’s mobility performance tests adjusted for a 4.572 m (15 ft) distance:	
Time ≥ 7 cm/s for height ≤ 159 cm or			Women: Time ≥ 5.6 cm/s (first quintile) for height ≤ 155.81 cm (average) or	
Time ≥ 6 cm/s for height > 159 cm			Women: Time ≥ 6.54 cm/s (first quintile) for height > 155.81 cm (average)	
			Men: Time ≥ 6.3 cm/s (first quintile) for height ≤ 169.55 cm (average) or	
			Men: Time ≥ 7.0 cm/s (first quintile) for height > 169.55 cm (average)	
**Weakness**	26,2%	20,8%	**Weakness**	20%
Grip strength ≤17 for BMI ≤23 kg/m^2^;			Martin Vigorimeter using the American Society of Hand Therapists procedure:	
Grip strength ≤17,3 for BMI 23,1–26 kg/m^2^;			Women: Grip strength ≤37.0 (first quintile) for BMI ≤23.49 kg/m^2^ (first quintile**)	
Grip strength ≤18 for BMI 26,1–29 kg/m^2^;			Women: Grip strength ≤35 .0 for BMI > 23.49 - ≤25.97 kg/m^2^ (second quintile)	
Grip strength ≤21 for BMI > 29 kg/m^2^;			Women: Grip strength ≤35.0 for BMI > 25.97 - ≤28.56 kg/m^2^ (third quintile)	
			Women: Grip strength ≤35.0 for BMI > 28.56 - ≤32.11 kg/m^2^ (fourth quintile)	
			Women: Grip strength ≤ 35.0for BMI > 32.11 kg/m^2^ (fourth quintile)	
			Men: Grip strength ≤47.4 (first quintile) for BMI ≤24.17 kg/m^2^ (first quintile)	
			Men: Grip strength ≤55.0 for BMI > 24.17 - ≤26.42 kg/m^2^ (second quintile)	
			Men: Grip strength ≤55.0 for BMI > 26.42 - ≤28.59 kg/m^2^ (third quintile)	
			Men: Grip strength ≤53.6 for BMI > 28.59 - ≤31.31 kg/m^2^ (fourth quintile)	
			Men: Grip strength ≤55.0 for BMI > 31.31 kg/m^2^ (fourth quintile)	
**Overall frailty status**			**Overall frailty status**	
Robust	33.2%	44,9%	Robust	49,7%
Pre-frail	55.2%	43,8%	Pre-frail	38,3%
Frail	11.6%	11,3%	Frail	12,0%

* Obtained from Bandeen-Roche et al. (2006), Table 1, page 263

** Quintiles were used for BMI to obtain wider ranges on grip strength

### Frailty components

With the FMM, continuous scores on the 5c-PMF components were used. Twelve respondents were not able to do the gait speed test. This component was considered left censured [20] and the twelve respondents were included in the analysis. Only 186 respondents lost weight. This component was defined as a Poisson with inflation variable [20]. Weight loss was thus represented with two items in the FMM with continuous variables: a dichotomous variable separating those with and those without weight loss, and a continuous variable with weight loss in kg for those losing weight (Table 1, last column).

Tests of the ability of the FRéLE data set to reproduce LCA PMF results used a priori dichotomized items obtained with the Bandeen-Roche et al. [3] procedures. Cut points were obtained separately for men and women. Differences in the operational definition of frailty components between the Women’s Health and Aging Studies (WHAS) and the FRéLE studies are found in Table 1. For clarity, only the CHS definitions are reproduced.

### Factors associated with frailty

Several variables were used to test predictive validity: Age and sex (RAMQ files); education (CCHS questions) [18]; self-reported health status (SF-36) [21]; number of chronic diseases (Functional Comorbidity Index (FCI)) [22]; depression symptoms (Geriatric Depression Scale (GDS)) [23]; cognitive functioning (Montreal Cognitive Assessment (MoCA)) [24]; disability in basic and instrumental activities of daily living (ADL, IADL) Katz et al., [25] Lawton and Brody [26] scales; mortality in the three-year period following the first interview obtained from the *Institut de la statistique du Québec*.

### Statistical analyses

Two of the conditions for the syndromic characterization of frailty are subcases of measurement-invariance conditions: K = 1-class and K > 1-categorical approximation of a continuous process [12]. These conditions are used as null hypotheses, as they impose homogeneity conditions on 5c-PMF measurement model parameters.

**Fig. 1:**
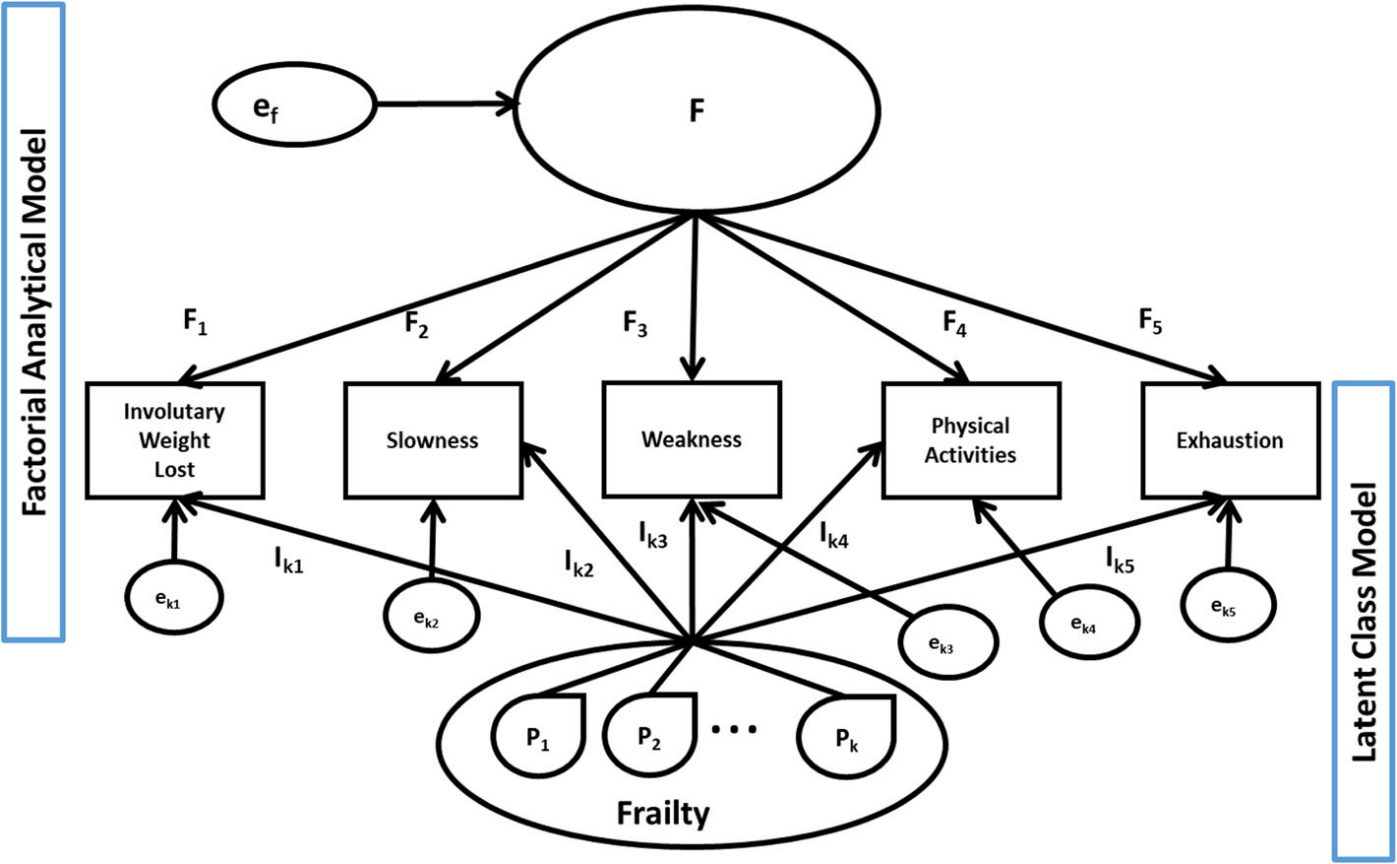
Factor Mixture Model (FMM). Structural parameters for syndromic frailty model with continuous measurements. Visual presentation of factorial analytical and latent class models]

Within LCA, the null hypothesis of homogeneity of a population is defined by a 1-class model. However, LCA can be considered a special case within a wider set of a family of models: factor mixture models (FMM). FMM tie a classification model (LCA) to a factor analytical model (FAM). Figure 1 shows a syndromic frailty FMM with continuous measurements. In the LCA (Fig. 1, lower part), FAM parameters are fixed to zero. Likewise, in FAM (Fig. 1, upper part), LCA parameters are excluded. In this case, the FMM parameters used to define the latent classes are null. Thus, the FAM is a model of homogeneity. Parameters generating latent classes are not defined within FAM, and FAM cannot be used to test the validity of a syndrome.

Measurement-invariance conditions operationalize a set of null and non-null models useful in studying syndromes. For practical reasons, Fig. 1 shows only the structural parameters of a syndromic frailty model with continuous measurements and only one factor with factor loadings invariant over classes. The I_kc_ (c = 1 to 5 frailty components in k = 1, …,K classes) are “intercepts”, or classification parameters. P_k_ (k = 1, …,K) classes are related to each of the “c” components through the I_kc_ intercepts. In FMM, part of the variation of the frailty components is used to classify individuals into frailty classes (classification parameters I_kc)_, and part (factor loadings F_c)_ is attributed to unmeasured constructs, among which are syndromes that share some of the frailty components. As represented in Fig. 1, factor loadings F_c_ for each “c” component do not vary between components. This factor structure can be said to be “reflective” [27, 28].. However, factor loadings in this “reflective” model are not used to classify individuals into frailty classes. Finally, in Fig. 1, residuals “e_kc_” are obtained on “c” components and one “e_f_” for the F factor.

Constraints over FMM parameters define seven basic FMM models; four of them correspond to null and three to non-null hypotheses on the syndromic characterization of frailty:

1. A one-class null model can be defined by two models:

- full FMM;
- LCA;

2. Two null hypotheses model are K > 1-categorical representation of a continuous process. Both imply that classification parameters and factor loadings are equal across classes:

- Strong measurement invariance (SoMI);
- Strict measurement invariance (SiMI). SiMI adds so SoMI equal residual variances across classes (Supplemental Material A);

3. Two non-null hypotheses are available. In both cases, classification parameters vary across classes. Both are compatible with the syndromic hypothesis for frailty inasmuch as the K = 1-class model is rejected. Both can be tested with residual variances, equal (ev) or unequal (uv) across classes:

- Weak measurement invariance (WMI-ev and WMI-uv);
- Null measurement invariance (NMI-ev and NMI-uv). In the NMI, factor loadings are unequal across classes;

4. LCA includes only the lower part of Fig. 1. LCA has equal (LCA-ev) or unequal (LCA-uv) residual variances. This is a non-null hypothesis as long as K > 1.

Given the non-rejection of LCA, WMI or NMI K > 1-class models, the ordering of the I_kc_ is compared among classes.

### Eight analytical steps

First, the 3-class 5c-PMF was tested on dichotomized components with FRéLE results compared to the Bandeen-Roche et al. results [3] on the WHAS. Second, the number of factorial dimensions in 5c-PMF components was examined with Confirmatory Factor Analysis (CFA) to design the FAM within the FMM. Third, null hypotheses of K = 1-class for frailty were examined for each of the FMM and LCA models using BIC and bootstrap likelihood ratio test (BLRT) statistics. BLRT was estimated by applying the Nylund et al. [29] procedure to 1000 Monte Carlo replications. Also, acceptability of the Monte Carlo parameter estimates was examined with the Muthén & Muthén [30] procedure (Supplemental Material B). The fourth step searched for the minimum acceptable K number of classes in each of the LCA and FMM models. Fifth, the hypotheses of uv were tested against the hypotheses of ev. Sixth, differences of classification parameters between classes, and their ordering, were examined in LCA and FMM models. Seventh, the null hypothesis of K > 1-categorical representation of a continuous process was tested against the selected non-null models (Supplemental Material C). And eighth, predictive validity was investigated in the 5c-PMF and in the final FRéLE model. All analyses were conducted using Mplus (Version 8) [20].

## Results

### Do FRéLE and WHAS results differ on LCA with dichotomous representation of frailty components?

Adding the frailty components that are met for each respondent, and grouping the scores in three frailty classes (robust: 0; prefrail: 1–2; frail: 3–4-5), frequencies of frail respondents in the WHAS, CHS [3] and FRéLE studies (Table 1) are almost the same (11.3 to 12.0%). The prefrail are a much larger proportion of respondents in the CHS (55.2%) than in the WHAS (43.8%) and the FRéLE (38.3%) data sets. FRéLE has the largest proportion of robust respondents.

Results from the LCA on FRéLE and WHAS dichotomous components are presented in Table 2. As expected, BIC for the 2-class models is smaller than BIC for the 1-class models. The BIC differences between the 3-class and 2-class models are positive, indicating a better fit for the latter. Also, with BIC, the hypothesis of no difference is not rejected in the comparison of 3-class with 2-class models for FRéLE and WHAS. However, the BLRT for FRéLE indicated a significant difference between the 3-class and the 2-class model, as in Lohman et al. [11]

**Table 2:** Latent Class Analysis (LCA): Results from the WHAS and the FRéLE studies

										Bootstrap
	**Loglikehood**			**Chi-Square Fit**		**BIC**		**Differences in BIC**		**loglikehood**
				***P*** **Values**						**ratio test**
**# of Classes**	**FRéLE**	**WASP**	**d.f.**	**FRéLE**	**WASP**	**FRéLE**	**WASP**	**FRéLE**	**WASP**	**FRéLE**
3 (vs. 2)	− 3665,2	N.Av.**	14	0,48	0,52	7456	3467	26	27	0,030
2 (vs. 1)	− 3674,1	N.A.	20	0,05	0,22	7430	3440	− 445	− 143	< 0.001
1 class	− 3919,0	N.A.	26	< 0.001	< 0.001	7875	3583	N.Ap.***	N.Ap.***	N.Ap.
* Woman Health and Aging Study										
** N.Av. Not Available										
*** N.Ap. Not Applicable										

LCA with dichotomized components on the FRéLE sample yielded the same frailty classes as in WHAS, though the WHAS participants were community-dwelling American women aged 70–79 years [3] while the FRéLE sample was drawn from community-living Canadians aged 65–93 years, mainly French-speaking. Also, measures for frailty components differed somewhat in the two studies (Table 1). Using LCA procedures with dichotomized items on the FRéLE sample, the null hypothesis of a homogeneous population (that is, frailty is not a syndrome) was rejected, making the FRéLE sample an acceptable starting point for revisiting the 5c-PMF even though some components were not measured in FRéLE on the same scales as the WHAS or the CHS.

### How many factors from the components?

In the second step, confirmatory FAM was run on two-factor and one-factor solutions. The likelihood ratio test (LRT) could not reject the null hypothesis of one dimension at α = 0.05 (X^2^ = 5.4 with 4 degrees of freedom) (Table not included).

### Is the null hypothesis of the 1-class model rejected in FRéLE with LCA and FMM models?

The null hypothesis of 1-class was rejected in both LCA and FMM according to BIC and BLRT (Table 3, lines 2 classes | 1 class in all subtables).

**Table 3:** Likelihood ratio-based tests for number of classes

					Bootstrap					
**k-class**			**Differences**		**1000 draws**	**Observed**	**Coefficients**	**S.E**	**Coverage**	**Power**
**Models**	**Entropy**	**BIC**	**in BIC**	**LL**	***p*** **= 0,05**	−2*(LL [H1]-LL [H0])	**Bias**	**Bias**		
**A: Latent classes: no factor loadings and no factor variance**										
**1. classes differ on intercepts [LCA-ev models]**										
4 classes***	N.A.**	N.A.	N.A.	N.A.	N.A.	N.A.	N.A.	N.A.	N.A.	N.A.
3 classes | 2 classes	0,681	65,379	−187	−32,611,7	17,5	231,4*	0,22%	1,58%	95,1%	99,9%
2 classes | 1 class	0,670	65,566	− 952	− 32,727,4	16,7	988,8*	0,18%	1,63%	95,0%	100,0%
1 class	N/A	66,518	N.A.	−33,221,8	N/A	N.A.	0,10%	1,58%	94,9%	99,1%
***Search process stopped. One class with case count equal to 14 only.										
**2. classes differ on intercepts and residual errors [LCA-uv models]**										
5 classes***	0,557	65,251	N.A.	−32,444,4	N.A.	N.A.	1,16%	17,2%	93,5%	96,7%
4 classes |3 classes	0,628	65,264	−62	−32,476,5	28,2	135,8*	0,55%	3,70%	94,3%	99,2%
3 classes | 2 classes	0,601	65,326	−156	−32,544,4	27,7	245,6*	0,39%	1,56%	94,4%	96,6%
2 classes | 1 class	0,696	65,482	− 1036	−32,667,2	31,5	1109,2*	0,15%	1,40%	95,1%	95,2%
1 class	N.A.	66,518	N.A.	−33,221,8	N.A.	N.A.	0,10%	1,58%	94,9%	99,1%
***S.E. bias greater than 10%										
**B: Factor mixture model - Classes do not differ on residual errors and factor variance**										
**1. Strict measurement invariance [SiMI-ev models]**										
3 classes***	N.A.	N.A.	N.A.	N.A.	N.A.	N.A.	N.A.	N.A.	N.A.	N.A.
2 classes | 1 class	0,219	65,359	10	−32,612,7	6,7	12,8*	2,98%	3,07%	94,9%	97,1%
1 class	N/A	65,349	N.A.	−32,619,1	N.A.	N.A.	0,89%	1,30%	95,1%	99,8%
***Search process stopped. One class with case count equal to one.										
**2. Weak measurement invariance [WMI-ev models]**										
4 classes****	N.A.	N.A.	N.A.	N.A.	N.A.	N.A.	N.A.	N.A.	N.A.	N.A.
3 classes! 1 classes	0,657	65,250	−99	−32,528,5	30,0	181,2*	1,08%	1,82%	94,9%	99,3%
2 classes***	0,743	65,281	N.A.	−32,566,5	N.A.	N.A.	0,32%	10,6%	94,6%	99,8%
1 class	N/A	65,349	N.A.	−32,619,1	N/A	N.A.	0,89%	1,30%	95,1%	99,8%
**** Search process stopped. One class with count equal to 18 only.										
***S.E. bias greater than 10%										
**3. No measurement invariance [NMI-ev models]**										
3 classes*** | 2 classes	0,484	65,264	−29	−32,506,2	24,8	103,2	1,75%	9,98%	95,1%	99,1%
2 classes | 1 class	0,709	65,293	−56	−32,557,8	26,0	122,6*	0,42%	1,82%	95,0%	96,6%
1 class	N.A.	65,349	N/A	−32,619,1	N.A.	N.A.	0,89%	1,30%	95,1%	99,8%
*** 3 classes model not significant										
**C: Factor mixture model - Classes differ on residual errors and factor variance**										
**1. Strong measurement invariance [SoMI-uv models]**										
5 classes***	N.A.	N.A.	N.A.	N.A.	N.A.	N.A.	N.A.	N.A.	N.A.	N.A.
4 classes | 2 classes	0,585	65,232	−32	−32,482,7	30,9	136,0*	0,96%	7,45%	93,6%	94,6%
3 classes***	0,437	65,234	N.A.	−32,506,1	N.A.	N.A.	0,71%	13,0%	94,4%	99,3%
2 classes | 1 class	0,420	65,264	−85	−32,550,7	18,1	136,8*	0,68%	2,03%	93,9%	92,9%
1 class	N.A.	65,349	N.A.	−32,619,1	N.A.	N.A.	0,89%	1,30%	95,1%	99,8%
***S.E. bias greater than 10%										
****										
**2. Week measurement invariance [WMI-uv models]**										
4 classes | 3 classes***	N.A.	N.A.	N.A.	N.A.	N.A.	N.A.	N.A.	N.A.	N.A.	N.A.
3 classes | 2 classes	0,393	65,202	−55	−32,456,9	27,1	143,0*	1,10%	6,43%	94,6%	99,0%
2 classes | 1 class	0,399	65,257	−92	−32,528,4	33,7	181,4*	0,88%	8,11%	94,6%	99,3%
1 class	N.A.	65,349	N/A	−32,619,1	N.A.	N.A.	0,89%	1,30%	95,1%	99,8%
*** The loglikelihood ratio for the (k-1) = 3-class model could not be computed. None of the coefficients on the factor part and of its variance were statistically significant. This model degenerated into the LPA 4-class model with equal variances										
**3. No measurement invariance [NMI-uv models]**										
4 classes**	N.A.	N.A.	N.A.	N.A.	N.A.	N.A.	N.A.	N.A.	N.A.	N.A.
3 classes | 1 classes	0,408	65,211	− 138	−32,438,8	58,4	360,6*	3,04%	9,8%	92,9%	94,1%
2 classes***	0,375	65,250	N.A.	−32,510,1	N.A.	N.A.	1,64%	10,7%	93,3%	99,1%
1 class	N.A.	65,349	N.A.	−32,619,1	N.A.	N.A.	0,89%	1,30%	95,1%	99,8%
** Search for a reduced model degenerated into the LPA model with 4 classes with unequal variances.										
***S.E. bias greater than 10%										
Note: * Null hypothesis rejected at p ≤ 0,05										
**N.A.: Not Applicable or Non Available										

### How many classes?

BLRT procedures and BIC statistics were used to identify the number of classes in each of the LCA and FMM models (see Supplemental Material D). Models and results for the Monte Carlo estimation procedure are shown in Table 3. All models met the Muthén and Muthén criteria [30]. In models that were not rejected, three to four classes were identified (Table 3).

### Are residuals equal over classes?

Within each model, the null hypothesis of equal variance (ev) over unequal variance (uv) was tested (Table 4, Parts A-D). Both BLRT and BIC tests rejected all equal variance models.

**Table 4:** Selecting the final frailty model

			# of		Differences		Bootstrap	Observed
	**# of Classes**	**Test**	**parameters**	**BIC**	**in BIC**	**LL**	**p = 0,05**	−2*(LL [H1]-LL [H0])
**A. Within LCA Models**								
**1. H [a.1]:LCA-uv**^**1**^	**4 classes**	**H [a.1]|H [a.0]**	42	65,264	115	−32,476,9	43,6	269,6*
**2. H [a.0]:LCA-ev**^**2**^	**3 classes**		21	65,379		−32,611,7		
**B. Within Strong Measurement Invariance (SMI) models**								
**3. H [b.1]:SoMI-uv**^**3**^	**4 classes**	**H [b.1]|H [b.0]**	36	65,232	120	−32,482,7	36,3	260,8*
**4. H [b.0]:SiMI-ev**^**4**^	**2 classes**		17	65,352		−32,613,1		
**C. Within Weak measurement invariance (WMI) models**								
**5. H [c.1]:WMI-uv**^**5**^	**3 classes**	**H [c.1]|H [c.0]**	38	65,189	61	−32,453,8	19,6	149,4*
**6. H [b.0]:WMI-ev**^**6**^	**3 classes**		26	65,250		−32,528,5		
**D. Within Null Measurement Invariance (NMI) models**								
**7. H [d.1]:NMI-uv**^**7**^	**3 classes**	**H [d.1]|H [c.0]**	45	65,211	82	−32,438,8	38,4	238,0*
**8. H [d.0]:NMI-ev**^**8**^	**2 classes**		25	65,293		−32,557,8		
**E. Testing the LPA-uv, WMI-uv and NMI-uv models against the null model (SMI-uv)**								
**9. H [d.1]:NMI-uv**^**7**^	**3 classes**	**H [d.1]|H [b.1]**	45	65,211	21	−32,438,8	282,3	87,8
**10. H [c.1]:WMI-uv**^**5**^	**3 classes**	**H [c.1]|H [b.1]**	38	65,189	43	−32,453,8	267,5	57,8
**11. H [a.1]:LPA-uv**^**1**^	**4 classes**	**H [a.1]|H [b.1]**	42	65,264	−32	−32,476,9	306,1	11,6
***The null model:***								
**12. H [b.1]:SMI-uv**^**4**^	**4 classes**		36	65,232	N.A.**	−32,482,7	N.A.	N.A.
***Notes:***								
* Null rejected								
** N.A. Not Applicable								
1. LPA-uv: LPA with unequal variances								
2. LPA-ev: LPA with equal variances								
3. SoMI-uv: FMM with Strong Measurement invariance - unequal variances								
4. SiMI-ev: FMM with Strict Measurement invariance - equal variances								
5. WMI-uv: FMM with Weak Measurement invariance - unequal variances								
6. WMI-ev: FMM with Weak Measurement invariance - equal variances								
7. NMI-uv: FMM with No Measurement invariance - unequal variances								
8. NMI-ev: FMM with No Measurement invariance - equal variances								

### Do frailty components increase in the same direction across the selected LCA, WMI and NMI classes?

In the 5c-PMF 3-class LCA models with dichotomized components, class thresholds within each component were different throughout classes, and were ordered in the same direction (Table 5, Part A). In the WMI-uv and NMI-uv, components were ordered on classes in two sets: 1. exhaustion, physical activity and weight loss as P1 → P2 → P3; 2. gait speed and grip strength as P2 → P1 → P3. The robust class is P3, while individuals with the lowest scores on gait speed and grip strength are located in P2. Individuals with greater weight loss, exhaustion and low physical activity are grouped in P1. The WMI-uv and NMI-uv models are thus excluded from further investigation as components are ordered differently. These two models suggest that the five components are from two syndromes: one indicative of muscle strength (gait speed and grip strength), the other may be an expression of exhaustion captured by three components: the perception of exhaustion (low scores on SF-36 vitality subscales), physical activity (low scores on the PASE), and weight loss. In the LCA-uv model, components are hierarchically ordered as expected.

**Table 5:** Factor loadings, class intercepts, class thresholds and classes means for the Fried, the SiMI and the WMI models

	Fried 3-Class model			SiMI 4-Class model				WMI 3-Class model			Total
	Frail	Pre-frail	Robust	Class 1	Class 2	Class 3	Class 4	Class 1	Class 2	Class 3	sample
**Part A. Coefficients for the Fried LCA 3-class, FRéLE SiMI-uv 4-classes and FRéLE WMI-uv models**											
**Factor loadings**											
**Exhaustion**	N.A.*	N.A.	N.A.	1,00	1,00	1,00	1,00	1,00	1,00	1,00	N.A.
**Physical Activities**	N.A.	N.A.	N.A.	4,10	4,10	4,10	4,10	4,61	4,61	4,61	N.A.
**Grip strength**	N.A.	N.A.	N.A.	1,21	1,21	1,21	1,21	1,13	1,13	1,13	N.A.
**Gait speed**	N.A.	N.A.	N.A.	2,17	2,17	2,17	2,17	3,38	3,38	3,38	N.A.
**Weight lost (in Kg)**	N.A.	N.A.	N.A.	−0,35	−0,35	−0,35	−0,35	−0,13	−0,13	−0,13	N.A.
**Class thresholds or Intercepts**											
**Exhaustion**	0,16	−0,82	− 2954	43,15	43,15	43,15	43,15	53,23	62,42	73,76	N.A.
**Physical Activities**	1,26	−0,92	− 7706	0,00	0,00	0,00	0,00	57,96	69,88	121,53	N.A.
**Grip strength**	0,38	−0,81	− 3293	34,77	34,77	34,77	34,77	56,44	47,73	73,53	N.A.
**Gait speed**	3,32	−1,05	− 3275	44,28	44,28	44,28	44,28	83,54	79,10	100,00	N.A.
**Weight lost (in Kg if > 0)**	N.A.	N.A.	N.A.	7,05	7,05	7,05	7,05	0,00	3,47	0,00	N.A.
**Weight lost (1 if Kg > 4,5; else = 0)**	1,05	1,27	3366	0,45	0,45	0,45	0,45	0,00	1,03	1,47	N.A.
**Means**	N.A.	N.A.	N.A.	32.232	21,72	8,53	0,00	N.A.	N.A.	N.A.	N.A.
**Part B. Associations of FRéLE and Fried classes with frailty components**											
**Means on components**											
**Exhaustion**	42,72	57,77	72,13	43,20	50,00	65,40	77,30	46,70	62,80	75,30	63,10
**Physical Activities**	11,07	67,92	111,92	−16,00	28,66	91,50	146,14	47,00	65,80	127,50	82,98
**Grip strength**	40,72	53,16	68,40	40,20	43,12	60,00	81,20	56,00	45,60	76,00	59,22
**Gait speed**	49,49	79,16	103,20	33,76	59,70	91,79	120,83	86,10	77,70	101,90	87,72
**Weight lost (in Kg if > 0)**	8,42	7,15	0,00	12,11	4,35	3,88	4,25	7,65	3,58	4,21	7,37
**Weight lost (1 if Kg > 0; else = 0)**	43,7%	21,0%	0,00%	38,6%	21,8%	9,5%	3,2%	23,7%	12,6%	3,6%	13,2%
**Part C. Associations of FRéLE classes and Fried classes with frailty risk factors**											
**Means on risk factors**											
**Age**	84,6	80,4	75,9	83,3	83,9	78,0	73,0	79,60	81,40	75,20	78,10
**Males**	46,8%	48,9%	51,2%	61,4%	32,5%	50,4%	69,5%	50,7%	29,6%	70,7%	50,0%
**Education**	4,41	5,22	5,72	4,18	4,55	5,48	6,30	5,14	4,93	5,99	5,37
**Self rated health**	3,44	2,93	2,34	3,43	3,23	2,64	2,06	3,16	2,82	2,25	2,71
**Chronic Diseases**	4,60	3,80	2,50	4,70	4,30	3,10	2,20	4,07	3,50	2,38	3,25
**Cognitive Impairment**	21,5	23,4	24,8	20,5	22,3	24,2	25,5	23,2	23,4	24,8	23,90
**Depressive symptoms**	62,9%	27,7%	7,4%	65,1%	45,3%	15,0%	5,2%	46,2%	21,9%	5,1%	22,1%
**Disability in IADL**	93,2%	55,4%	23,4%	100,0%	85,4%	34,8%	8,8%	62,8%	58,0%	1,6%	44,4%
**Disability in ADL**	66,8%	30,3%	11,3%	84,1%	49,5%	17,7%	7,1%	39,6%	30,5%	10,0%	25,6%
**Deceased**	34,8%	14,5%	5,9%	40,9%	22,9%	9,5%	4,9%	20,7%	13,7%	6,3%	13,0%
**Number of cases**	207	627	807	47	411	874	309	422	628	591	1641
**% in each class or class**	12,5%	38,3%	49,2%	2,7%	25,1%	53,4%	18,8%	25,7%	38,3%	36,0%	

*N.A.: Not Applicable

### Is the null hypothesis of strong measurement invariance (SoMI) rejected?

The LCA-uv model was tested against the null hypothesis represented by the SoMI model (Table 4, Part E). With both the BIC and BLRT, the SoMI model could not be rejected. That is, the null hypothesis that the population is homogeneous (suggesting that the 5c-PMF is not a measure of frailty as a syndrome) could not be rejected in the final model.

### Are SoMI-uv classes associated with expected predictors and consequences of frailty?

Even though SoMI-uv classes yielded factor loadings and class intercepts of equal value within components (Table 5, Part A), they are distributed along a single, ordered latent variable.

Distributions of frailty components in the FRéLE SoMI 4-class, with continuous components, and the 5c-PMF LCA 3-class models, with dichotomized components, are shown in Table 5, Part B. Components at baseline were distributed as expected in the 5c-PMF LCA; their values increased from frail to robust classes in each case. The SoMI model replicated these results, except for involuntary weight loss in kilograms. Also, in all cases, the range of variation of components between classes was greater in the SoMI than in the 5c-PMF LCA. The results were replicated with factors associated with frailty at T2 (Table 5, Part C), except for gender. There were no gender differences between frailty classes in the 5c-PMF LCA. However, the SoMI model showed that males represent 61.4% of the lowest health status class.

The SoMI-uv classes were associated with the five frailty components at a higher level than the 5c-PMF. These classes were also associated with some of the socio-economic and health status variables usually considered in studies of predictive validity for frailty, even though they were generated from a categorization of a continuous latent variable.

## Discussion

The aim of this study was to examine the validity of the measurement of the syndrome of frailty based on the five components of the PMF using FMM. Our results show that the hypothesis of homogeneity, indicating the inability to distinguish between frailty classes based on the 5c-PMF, cannot be rejected.

The 5c-PMF is a measure of frailty as a syndrome, if its components identify at least two classes among a population: those with the syndrome, and those without the syndrome. Thus, models used to investigate the 5c-PMF must test parameters generating frailty classes. Statistical models that do not have parameters that generate classes, or do not offer tests on classification parameters, are not appropriate.

The FMM framework includes classification parameters and allows testing three null homogeneity hypotheses: 1. K > 1-categorical representation of a continuous process; 2. K = 1-class mode; 3. A model with subsets of components occurring in only some of the classes. All of these hypotheses are defined in terms of classification parameters. Inasmuch as two or more well-separated classes are obtained, each component will also show well-separated distributions within each class. The FMM procedure classifies observed cases according to their scores on each of the components. Also, cut points for each component can be identified. Classification parameter estimates and cut points may be obtained from different samples and their values compared (Supplemental Material E). Thus, the FMM framework can take into account variations of the manifestation of syndromes in different contexts.

In our study, one of the three null hypotheses could not be rejected – the SoMI-uv model. In effect, the estimated classification parameters in this model are equal across classes for each frailty component, the distribution of components within classes is not well-separated, and cut points appear arbitrary (Supplemental Material E). They do not meet the Bandeen-Roche et al. [3] requirement for syndromic frailty which specifies that parameters are different and ordered similarly between each class.

Variations of frailty components in categories of the SoMI-uv model were wider in scope than variations in the PMF’s robust, prefrail and frail classes. Similar results were obtained with factors (measured at T2) associated with frailty classes (measured at T0). Thus, predictive validity of the 4-categorical representation of frailty as a continuous process was higher than the predictive validity of the 5c-PMF. This suggests that a model generating frailty categories by a continuous process (the SoMI-uv model) may be a useful health status construct in population health surveys, even though frailty cannot be considered a syndrome. The 4-categorical representation of frailty is the best categorization of frailty as a continuous variable in the FRéLE study. Other health status constructs, such as self-rated health, though not a syndrome, have proven to be useful in population health studies. However, the validity and reliability of a continuous construct of frailty using the five PMF components need to be examined using recognized psychometric techniques. Reflective and / or formative models [27, 28] may offer useful conceptual schemes and operational procedures to examine the construct of frailty as a continuous variable. This examination was beyond the scope of our study.

There are a number of limitations to this study:

1. Some of the measurements of component in FRéLE were the same as in Bandeen-Roche et al., [3] while others differed. However, LCA with dichotomized components conducted on the FRéLE sample replicated the results reported in WHAS [3];
2. Frailty components represent specific points in the frailty biological cycle [1]. On the one hand, they are manifestations of a clinical syndrome [1]. On the other hand, etiological and pathophysiological processes are at the source of clinical manifestations [8]. Given the results of our analysis, frailty based on the five continuous components of the PMF cannot be used to classify individuals in classes of frailty as a syndrome. The refinement of measures that use biological bases for frailty may lead to a stronger theoretical rational for sound clinical measures [31];
3. The five-component model used in PMF to measure frailty is a clinical construct. The 4-categorical representation of frailty as a continuous process (the SoMI model), applies only to the clinical characterization of frailty with the 5c-PMF.This conclusion cannot be extended to the validity of the pathophysiological and etiological processes of frailty, as represented in the frailty cycle.
4. One of the SoMI-uv model classes is small. This class represents individuals with low scores from all PMF components. In a larger sample or in a sample having a lower average health and physical function than the FRéLE cohort, the separation of this group from the other three may become significant enough to break the continuity of the categorical representation of the continuous process found in our study. Thus, replication of this study is needed in international cohorts with a wide range of physical function and survival rates.

## Conclusion

Inasmuch as frailty is a syndrome, the frailty measure used in clinical settings or in population health surveys should at the very least differentiate individuals with the syndrome from those without the syndrome [3, 8]. However, the predictive ability of a syndrome depends on a clear understanding of the notion of syndrome, on the modelling of its etiological factors and pathophysiological processes, and how its clinical components are linked with these processes. We have shown that the association of categories based on a single continuum of frailty (SoMI model categories) with a set of well-known correlates of adverse outcomes in old age was as strong as, or stronger than, with 5c-PMF categories. This is an example of the caution needed in using predictive validity to examine the validity of frailty measurements. Controlling for age, sex, and chronic disease, other studies have shown a specific but weak contribution of frailty to disability [32]. These results are an illustration of the Xue et al. [6] injunction “… to move beyond predictive validity to examine consistency of frailty diagnosis and its implication …” .Without these features, addressing frailty in a clinical setting may not improve patient health [33]. Thus, research may have to focus on frailty as a biological entity, examine its etiological and pathophysiological basis and its consistency as a diagnosis. Without a sound basis, the search for valid and reliable measurement tools for public health and clinical practice may be a frustrating endeavor that produces a collection of measures [34] of detrimental states resulting in detrimental consequences.

## Supplementary Information

**Additional file 1 Supp A** Strict and strong measurement invariance. Describes statistical concepts.

**Additional file 2 Supp B** Montecarlo acceptability tests. Describes statistical concepts

**Additional file 3 Supp C** Analytical steps. Further description of analytical strategy

**Additional file 4 Supp D** Choosing K-class LPA and FMM models. Describes model selection

**Additional file 5 Suppl E** (with companion Supp Figs. E). Cut points with components measured on continuous scales. Examples of poor and good separation between classes

## Data Availability

The datasets used and/or analysed during the current study are available from the corresponding author on reasonable request.

## Abbreviations

5c-PMF: 5 components Phenotype Model of Frailty
ADL: Activity of Daily Living
BIC: Bayesian Information Criterion
BLRT: Bootstrap Likelihood Ratio Test
CCHS: Canadian Community Health Survey
CFA: Confirmatory Factorial Analysis
CHS: Caridiovascular Health Study
ev: equal variance
FAM: Factor Analytical Model
FCI: Functional Comorbidity Index
FFM: Factor Mixture Model
FRéLE: Fragilité: une étude longtitudinale de ses expressions
GDS: Geriatric Depression Scale
IADL: Instrumental Activity of Daily Living
LCA-ev: Latent Class Analysis – equal variance
LCA-uv: Latent Class Analysis– unequal variance
LCA: Latent Class Analysis
LRT: Lidelihood Ratio Test
MoCA: Montreal Cognitive Assessment
NMI: Null Measurement Invariance
NMI-ev: Null Measurement Invariance – equal variance
NMI-uv: Null Measurement Invariance – unequal variance
PASE: Physical Activity Scale for the Elderly
PMF: Phenotype Model of Frailty
RAMQ: Régie de l’assurance maladie du Québec
SiMI: Strict Measurement Invariance
SoMI: Strong Measurememtn Invariance
uv: unequal variance
WHAS: Women’s Health and Aging Study
WMI: Weak Measurement Invariance
WMI-ev: Weak Measurement Invariance – equal variance
WMI-uv: Weak Measurement Invariance – unequal variance
