# Supplementary material for: Revisiting the hypothesis of syndromic frailty: a cross-sectional study of the structural validity of the frailty phenotype
Source: BMC Geriatr. 2020 Oct 27;20:429. doi: 10.1186/s12877-020-01839-7 (PMC7590708; doi:10.1186/s12877-020-01839-7)
Supplement: Supplementary file 1 — Additional file 1 Supp A Strict and strong measurement invariance. Describes statistical concepts. [file 12877_2020_1839_MOESM1_ESM.docx]

**Supplemental Material A: Strict and strong measurement invariance**

Residual variances include two indistinguishable components in FMM: a random part and a systematic part. The random part is unproblematic. ([1](#_ENREF_1)) The systematic part is attributable to variables excluded from the analysis. With unequal systematic residuals, excluded variable associations with class components may also vary. Thus, SoMI solutions with more than one class may stem from variables other than, but associated with, frailty components. However, in the Bandeen-Roche et al. ([2](#_ENREF_2)) theoretical framework, frailty classes are obtained from frailty components, not from variables associated with them. This is the case of Lubke and Muthén ([3](#_ENREF_3)) where strong theoretical reasons are present for not rejecting measurement invariance with the SoMI model.

**References**

1. Lubke GH, Dolan CV. Can Unequal Residual Variances Across Groups Mask Differences in Residual Means in the Common Factor Model? Structural Equation Modeling: A Multidisciplinary Journal. 2003;10(2):175-92.

2. Bandeen-Roche K, Xue Q-L, Ferrucci L, Walston J, Guralnik JM, Chaves P, et al. Phenotype of frailty: characterization in the women's health and aging studies. The Journals of Gerontology Series A: Biological Sciences and Medical Sciences. 2006;61(3):262-6.

3. Lubke GH, Muthen B. Investigating population heterogeneity with factor mixture models. Psychol Methods. 2005;10(1):21-39.
