# Supplementary material for: Revisiting the hypothesis of syndromic frailty: a cross-sectional study of the structural validity of the frailty phenotype
Source: BMC Geriatr. 2020 Oct 27;20:429. doi: 10.1186/s12877-020-01839-7 (PMC7590708; doi:10.1186/s12877-020-01839-7)
Supplement: Supplementary file 2 — Additional file 2 Supp B Montecarlo acceptability tests. Describes statistical concepts [file 12877_2020_1839_MOESM2_ESM.docx]

**Supplemental Material B: Montecarlo acceptability tests**

Results of the acceptability tests are found in Table 2 and Supplemental Table B. Only averages over coefficient and residual variance biases, as well as coverage and power, are listed. Results for each of the parameters in each model require unwieldy tables. In a few cases, residual variance biases and coverage indices for specific parameters fell outside of the acceptable range. These cases are mentioned where appropriate.

Coverage indices for full models are provided in Table B, column 2. A few were within the 92-98% range, while all of the restricted model indices were within range. The null hypothesis of no difference between the full and restricted models with 1-class model is rejected in all of the FMM models with a chi-square of 4.4, while the 0.05 p-level is 3.84 with 1 degree of freedom. However, all the BIC for the full models are larger than the BIC for restricted models. The null hypothesis of no difference between the full and restricted models was rejected with the LRT for LPA models, except for the 1-class and 4-class unequal variance models. Finally, the null-hypothesis of no difference was not rejected in only two of 12 cases in the FMM models according to the LRT. However, the BIC was positive in only one case.

Average bias indices for model coefficients were quite small (Table 2). Power was uniformly high. Though averages for coverage indices were within the acceptable range, some sets of coverage indices for specific parameters in some classes were lower than expected (data not shown). This usually occurred in models with biases in variances higher than the 10% threshold. Models with biases over the threshold for variances were excluded in the BLRT procedure as they introduced instability in the bootstrap estimates. ([1](#_ENREF_1))

**References**

1. Muthén LK, Muthén BO. How to Use a Monte Carlo Study to Decide on Sample Size and Determine Power. Structural Equation Modeling: A Multidisciplinary Journal. 2002;9(4):599-620.

**Supplemental Table B. Selected reduced models and average estimated parameter biases, coverage and power**

|  | **Full Models[H0]** | | | **Reduced Models[H1]** | | | **LRT test[H0\|H1]** | |  |
| --- | --- | --- | --- | --- | --- | --- | --- | --- | --- |
| **k-Class** | **LL[H0]** | **Coverage** | **BIC[H0]** | **LL[H1]** | **Coverage** | **BIC[H1]** | **Differences** | **degrees** | **Difference** |
| **Models** |  |  |  |  |  |  | **in LL** | **of freedom** | **in BIC** |
|  | **-1** | **-2** | -3 | **-4** | **-5** | -6 | **-7** | -8 | **-9** |
| **A: Latent classes: no factor loadings and no factor variances** | | | | |  |  |  |  |  |
| **1. Classes differ on intercepts** | | |  |  |  |  |  |  |  |
| 3 classes | -32603,8 | 79,70% | 65392 | -32611,7 | 95,10% | 65379 | 15,8* | 4 | -13 |
| 2 classes | -32714,9 | 84,50% | 65563 | -32727,4 | 95,00% | 65566 | 25,0* | 3 | 3 |
| 1 class | -33221,2 | 87,90% | 66524 | -33221,8 | 94,90% | 66518 | 1,2 | 1 | -6 |
|  |  |  |  |  |  |  |  |  |  |
| **2. Classes differ on intercepts and residual errors** | | | | | | | | | |
| 4 classes | -32472,2 | 52,90% | 65292 | -32476,9 | 94,40% | 65264 | 9,4 | 5 | -28 |
| 3 classes | -32530,4 | 80,80% | 65320 | -32544,4 | 94,40% | 65326 | 28,0* | 3 | 6 |
| 2 classes | -32655,0 | 89,50% | 65480 | -32667,2 | 95,10% | 65482 | 24,4* | 3 | 2 |
| 1 class | -33221,2 | 87,90% | 66524 | -33221,8 | 94,90% | 66518 | 1,2 | 1 | -6 |
|  |  |  |  |  |  |  |  |  |  |
| **B: Factor mixture model - Classes do not differ on residual errors and factor variances** | | | | | | |  |  |  |
|  |  |  |  |  |  |  |  |  |  |
| **1. Strict measurement invariance** | | |  |  |  |  |  |  |  |
| 2 classes | -32612,7 | 86,60% | 65359 | -32613,1 | 94,90% | 65352 | 0,8 | 1 | -7 |
| 1 class | -32616,9 | 92,50% | 65352 | -32619,1 | 95,10% | 65349 | 4,4* | 1 | -3 |
|  |  |  |  |  |  |  |  |  |  |
| **2. Weak measurement invariance** | | |  |  |  |  |  |  |  |
| 4 classes | -32501,6 |  | 65277 | -32514,1 | 94,70% | 65243 | 25,0* | 8 | -34 |
| 3 classes | -32523,9 | 88,10% | 65270 | -32528,5 | 94,90% | 65250 | 9,2 | 4 | -20 |
| 2 classes | -32562,8 | 90,50% | 65296 | -32566,5 | 94,60% | 65281 | 7,4 | 3 | -15 |
| 1 class | -32616,9 | 92,50% | 65352 | -32619,1 | 95,10% | 65349 | 4,4* | 1 | -3 |
|  |  |  |  |  |  |  |  |  |  |
| **3. No measurement invariance:** | | |  |  |  |  |  |  |  |
| 3 classes | -32502,1 | 91,10% | 65286 | -32506,2 | 95,10% | 65264 | 8,2 | 8 | -22 |
| 2 classes | -32555,1 | 92,10% | 65310 | -32557,8 | 95,00% | 65293 | 5,4 | 3 | -17 |
| 1 class | -32616,9 | 92,50% | 65352 | -32619,1 | 95,10% | 65349 | 4,4* | 1 | -3 |
|  |  |  |  |  |  |  |  |  |  |
| **C: Factor mixture model - Classes differ on residual errors and factor variances** | | | | | | |  |  |  |
|  |  |  |  |  |  |  |  |  |  |
| **1. Strong measurement invariance** | | |  |  |  |  |  |  |  |
| 4 classes | -32482,4 | 88,60% | 65261 | -32482,6 | 93,80% | 65232 | 0,4 | 4 | -29 |
| 3 classes | -32503,1 | 91,10% | 65250 | -32506,1 | 94,40% | 65234 | 6,0* | 2 | -16 |
| 2 classes | -32548,3 | 93,20% | 65270 | -32550,7 | 93,90% | 65264 | 4,8 | 2 | -6 |
| 1 class | -32616,9 | 92,50% | 65352 | -32619,1 | 95,10% | 65349 | 4,4* | 1 | -3 |
|  |  |  |  |  |  |  |  |  |  |
| **2. Week measurement invariance** | | |  |  |  |  |  |  |  |
| 3 classes | -32448 | 82,50% | 65207 | -32456,9 | 94,60% | 65202 | 17,8* | 3 | -5 |
| 2 classes | -32527,7 | 27,30% | 65270 | -32528,4 | 94,60% | 65257 | 1,4 | 2 | -13 |
| 1 class | -32616,9 | 92,50% | 65352 | -32619,1 | 95,10% | 65349 | 4,4* | 1 | -3 |
|  |  |  |  |  |  |  |  |  |  |
| **3. No measurement invariance** | | |  |  |  |  |  |  |  |
| 3 classes | -32436,2 | 91,90% | 65243 | -32438,8 | 92,90% | 65211 | 5,2 | 5 | -32 |
| 2 classes | -32510,1 | 87,50% | 65265 | -32510,1 | 93,30% | 65250 | 0 | 2 | -15 |
| 1 class | -32616,9 | 92,50% | 65352 | -32619,1 | 95,10% | 65349 | 4,4* | 1 | -3 |
|  |  |  |  |  |  |  |  |  |  |
| * Null hypothesis rejected at p ≤ 0,05. | | |  |  |  |  |  |  |  |
