# Supplementary material for: Revisiting the hypothesis of syndromic frailty: a cross-sectional study of the structural validity of the frailty phenotype
Source: BMC Geriatr. 2020 Oct 27;20:429. doi: 10.1186/s12877-020-01839-7 (PMC7590708; doi:10.1186/s12877-020-01839-7)
Supplement: Supplementary file 3 — Additional file 3 Supp C Analytical steps. Further description of analytical strategy [file 12877_2020_1839_MOESM3_ESM.docx]

**Supplemental Material C: Analytical steps**

*First step:* Prevalence of frailty in FRéLE, CHS, ([1](#_ENREF_1)) and WHAS ([2](#_ENREF_2)) samples was compared. FRéLE participants were categorized into robust, pre-frail, and frail using Fried et al ([1](#_ENREF_1)) criterion. LCA was conducted using the Bandeen-Roche et al. ([2](#_ENREF_2)) framework and statistical procedure. The BIC criteria and chi-square statistics were used to test for frailty as syndromic. The Mplus BLRT ([3](#_ENREF_3)) was also run on the FRéLE data set. FRéLE and WHAS LCA results were compared.

*Second step:* The number of factorial dimensions was examined with CFA using LRT and BIC on the FRéLE sample continuous frailty component scores. Factor analysis parameter estimates were entered as starting values in FMM tests.

*Third step:* The null hypothesis of 1-class for frailty was examined in both the FMM and LPA models. Using the BIC and BLRT statistics, the k=1,…K class models were compared with k-1 models. The BLRT tests were obtained applying the Nyland et al. ([4](#_ENREF_4)) procedure with 1,000 Montecarlo replications. As usual in chi-square tests, twice the difference in the LL of the null-hypothesis with the LL of the tested models is compared with the LL 0.05 p-level obtained from the Montecarlo procedure. The quality of Montecarlo generated parameter estimates, residual variances, parameters coverage, and power was examined. ([5](#_ENREF_5)) Muthén and Muthén ([3](#_ENREF_3)) criteria were used: parameter and residual variance biases were fixed at 10% or lower, 95% confidence interval coverage was tolerated within a 0.91 – 0.98 range; power was set at 0.80 and higher. FMM and LPA models, with all parameters included (full model) were tested. If quality criteria were not respected with full models, acceptable restricted models were generally obtained from examination of full model parameter estimates. Some of the parameters were not statistically significant at the 0.05-levels; others had low power (<0.80). These parameters were excluded to obtain the restricted models. The null hypothesis of no difference between the full model and the restricted model was examined with LRT and BIC tests. A restricted model was selected if the null hypothesis could not be rejected with the LRT and/or if the restricted model BIC was equal or lower than the full model BIC. If an acceptable restricted model could not be obtained, the model was considered unfit and excluded from further testing.

*Fourth step:* Inasmuch as the null hypothesis of K=1 was rejected, the search began for the minimum acceptable K number of classes. BLRT and BIC were used to select K classes. However, analysis was ended if a class had a small number of cases, if the BLRT failed to converge, or if factor loadings lost statistical significance, degenerating into an LPA model.

*Fifth step:* Within each LPA, WMI and NMI model types, the null hypothesis of ev was tested against the uv models using the BLRT and BIC. The SiMI model was tested against the SoMI model. They differed to the extent that component residual variances were equal or not throughout components.

*Sixth step:* The null hypothesis of K>1-categorical representation of a continuous process was tested. The SoMI or SiMI of the previous step was considered as null hypothesis and tested against the selected LPA, WMI and NMI models.

*Comparison between frailty classes of the selected model*

Association of frailty classes from the final selected model with socio-demographic (age and sex), socio-economic (education) and health status (self-perceived health, number of chronic diseases, depression, cognitive status, and ADL and IADL) was examined. Classes with higher values on frailty components were expected to generate higher frequencies for older women with lower educational and income levels, and poorer health status than robust or pre-frail classes. Component mean scores between classes were also obtained to estimate class separation among components.

**References**

1. Fried LP, Tangen CM, Walston J, Newman AB, Hirsch C, Gottdiener J, et al. Frailty in Older Adults: Evidence for a Phenotype. The Journals of Gerontology: Series A. 2001;56(3):M146-M57.

2. Bandeen-Roche K, Xue Q-L, Ferrucci L, Walston J, Guralnik JM, Chaves P, et al. Phenotype of frailty: characterization in the women's health and aging studies. The Journals of Gerontology Series A: Biological Sciences and Medical Sciences. 2006;61(3):262-6.

3. Muthén LK, Muthén BO. Mplus User’s Guide. Eight ed. Los Angeles, CA: Muthén & Muthén; 1998-2017.

4. Nylund KL, Asparouhov T, Muthén BO. Deciding on the Number of Classes in Latent Class Analysis and Growth Mixture Modeling: A Monte Carlo Simulation Study. Structural Equation Modeling: A Multidisciplinary Journal. 2007;14(4):535-69.

5. Muthén LK, Muthén BO. How to Use a Monte Carlo Study to Decide on Sample Size and Determine Power. Structural Equation Modeling: A Multidisciplinary Journal. 2002;9(4):599-620.
