# Supplementary material for: Revisiting the hypothesis of syndromic frailty: a cross-sectional study of the structural validity of the frailty phenotype
Source: BMC Geriatr. 2020 Oct 27;20:429. doi: 10.1186/s12877-020-01839-7 (PMC7590708; doi:10.1186/s12877-020-01839-7)
Supplement: Supplementary file 4 — Additional file 4 Supp D Choosing K-class LPA and FMM models. Describes model selection [file 12877_2020_1839_MOESM4_ESM.docx]

**Supplemental Material D: Choosing K-class LPA and FMM models**

In five out of eight model types (Table 3), the small number of cases in one of the classes ended the testing procedure for added classes. With case counts varying from one to 18 and with six to ten parameters to estimate within each class, applying the BLTR procedure introduced instability in the estimation procedures. Also, for the SoMI-uv model, the 5-class restricted model did not reach all of the quality criteria for applying BLRT.

The BLRT and BIC procedures provided statistically-based stopping rules for two model types only. The NMI-uv 3-class model with equal variances was rejected against the NMI-uv 2-class model (Table 3, B.3). Using BIC, the SiMI-ev 2-class and 1-class models were not different (Table 3, B.1). However, the BLRT test suggested rejecting the null hypothesis of no difference. In brief, the number of classes varied from two to four in selected models.
