# Supplementary material for: Revisiting the hypothesis of syndromic frailty: a cross-sectional study of the structural validity of the frailty phenotype
Source: BMC Geriatr. 2020 Oct 27;20:429. doi: 10.1186/s12877-020-01839-7 (PMC7590708; doi:10.1186/s12877-020-01839-7)
Supplement: Supplementary file 5 — Additional file 5 Suppl E (with companion Supp Figs. E). Cut points with components measured on continuous scales. Examples of poor and good separation between classes [file 12877_2020_1839_MOESM5_ESM.docx]

**Supplemental Material E: Cut points with components measured on continuous scales**

In this study, except for the comparison of the PMF in the WHAS and FRéLE studies, continuous measurements of frailty components were entered in the FMM. Cut points were not defined a priori. Inasmuch as the FMM results would have shown good separation between classes, cut points would have resulted from the analysis. However, our study concluded that the classification parameters were equal in each class suggesting there was no separation between frailty classes. An example may clarify this point.

Inasmuch as frailty is a syndrome, the odds are that well-separated mixed distributions of continuous frailty components will be obtained with FMM. Also, well-defined classes with small variations of component scores within each class will be identified.

Examples of poorly separated classes can be generated using our results from the K>1-categorical approximation of a continuous latent variable for frailty. The separation between classes for grip strength is shown in Figure 1E. For the sake of clarity, only the three largest classes are considered. Clearly, estimated grip strength distributions for each class largely overlap; there is no evidence for well-separated classes of frail, prefrail and robust respondents, and no clear cut points appear. In contrast, Figure 2E shows a simulation of well-separated classes that could have been obtained from a model not rejecting syndromic frailty. In Figure 2E, overlap between classes is minimal, suggesting observed cutting points over grip strength.

**Figure 1E. Continuous distributions of grip strength for three latent classes from the K>1-categorical model.**

**Figure 2E. Well-separated frailty classes: Simulated distributions of continuously measured grip strength.**
